# Durable cytotoxic immune responses against gp120 elicited by recombinant SV40 vectors encoding HIV-1 gp120 ± IL-15

**DOI:** 10.1186/1479-0556-2-10

**Published:** 2004-08-23

**Authors:** Hayley J McKee, Patricia Y T'sao, Maria Vera, Puri Fortes, David S Strayer

**Affiliations:** 1 Department of Pathology, Jefferson Medical College, Philadelphia, PA, USA; 2 School of Medicine. Foundation for Applied Medical Research. Division of Gene Therapy. Laboratory of Vector Development. University of Navarra. Irunlarrea 1. 31008. Pamplona. Spain

**Keywords:** SV40, HIV-1 gp120, IL-15, cytotoxic memory

## Abstract

**Background:**

A vaccine that elicits durable, powerful anti-HIV immunity remains an elusive goal. In these studies we tested whether multiple treatments with viral vector-delivered HIV envelope antigen (gp120), with and without IL-15, could help to approach that goal. For this purpose, we used recombinant *Tag*-deleted SV40-derived vectors (rSV40s), since they do not elicit neutralizing antibody responses, and so can be given multiply without loss of transduction efficiency.

**Methods:**

SV(gp120) carried the coding sequences for HIV-1NL4-3 Env, and SV(mIL-15) carried the cDNA for mouse IL-15. Singly, and in combination, these two vectors were given monthly to BALB/cJ mice. Cytotoxic immunity and cytotoxic memory were tested in direct cytotoxicity assays using unselected effector cells. Antibody vs. gp120 was measured in a binding assay. In both cases, targets were P815 cells that were stably transfected with gp120.

**Results:**

Multiple injections of SV(gp120) elicited powerful anti-gp120 cytolytic activity (>70% specific lysis) by unselected spleen cells. Cells from multiply-immunized mice that were rested 1 year after their last injections still showed >60% gp120-specific lysis. Anti-gp120 antibody was first detected after 2 monthly injections of SV(gp120) and remained elevated thereafter. Adding SV(mIL-15) to the immunization regimen dramatically accelerated the development of memory cytolytic responses, with ≥ 50% specific lysis seen 1 month after two treatments. IL-15 did not alter the development of antibody responses.

**Conclusions:**

Thus, rSV40s encoding antigens and immunostimulatory cytokines may be useful tools for priming and/or boosting immune responses against HIV.

## Introduction

The development of an effective vaccine against HIV has been hindered by a variety of problems. The high mutation rate of the virus itself is such that it represents a moving antigenic target during the course of an infection [1-4]. Furthermore, HLA-A and -B expression is directly downregulated by HIV (via intracellular blocking of class I MHC-export to the cell surface by HIV-1 Nef and Vpu), so that efficient antigen presentation is compromised [1,5].

Compared to administration of protein antigen or naked DNA, an infectious vector could be more effective at enhancing antibody and cytotoxic responses against a transgene product. Application of such a strategy, however, has been often complicated by the development of neutralizing immune responses, principally antibodies, against vector coat antigens [6-10]. These neutralizing antibodies arise because the viral vectors enter cells largely through endocytic pathways. Their capsids, like most other particulate antigens, are processed at the time of infection and presented to the immune system. Resulting immune responses neutralize subsequent injections of the vector, and so limit the ability of that vector to be used repeatedly to boost immune responses.

This limitation can be circumvented by repeatedly changing the serotype of the antigen-carrying vector, or by using recombinant *Tag*-deleted SV40-derived gene delivery vectors (rSV40s) for immunization. Several studies have shown, both directly and indirectly, that rSV40 vectors do not elicit detectable neutralizing antibodies [11-13]. Even repeated administration of single [11,12] or different [13] rSV40 vectors in normal, immunocompetent hosts does not generate antibodies against the vector capsid proteins sufficiently to impair the ability of these vectors to deliver their genes efficiently *in vivo*.

The explanation for this unusual state of affairs may lie in the fact that SV40 enters cells via caveolae and thence travels directly to the nucleus, bypassing cellular antigen processing [14-16]. Thus, only proteins expressed by virus can elicit immune responses. Since, for *Tag*-deleted rSV40 vectors (unlike wild type SV40), capsid proteins are not expressed, immune responses can only be generated by transgene products [11,12]. Whether for this or for other reasons, rSV40 vectors can be used multiple times to prime and/or boost immune responses against antigens encoded by the transgenes they carry [13,14]. We have previously shown that powerful transgene-specific cytolytic and serum antibody responses can be detected in mice inoculated with rSV40 carrying the cDNA for SIVmac239 envelope glycoprotein gp130 [12]. Four to five monthly immunizations were adequate to produce >50% specific lysis of envelope-expressing target cells, even with effector:target ratios of 10:1 [12].

Other investigators have reported that co-administration of vectors carrying immunostimulatory cytokines was useful in augmenting anti-lentiviral immune responses [17-19]. IL-15 has various immunostimulatory and immunomodulatory effects, among which is the ability to upregulate activated T cell proliferation and induce cytotoxic T cell activity [20]. It also promotes cytotoxic T cell memory [21,22].

Both antibody and cell-mediated immune responses may be useful to protect from HIV infection and progression to AIDS [23-26]. However, there is a particularly good correlation between long-term non-progression to AIDS and strong CTL responses in HIV-positive individuals [22,27-31]. Weak CTL responses are generally seen in those who progress rapidly to disease, and in children. Because of the importance of a virus-specific cytotoxic T cell (CTL) response, one of the major aims of any vaccine should be to elicit strong HIV-specific CTL responses [32,33].

We used rSV40s to study the generation and longevity of both humoral and cell-mediated responses in an effort to generate immune responses against the HIV-1 envelope glycoprotein, gp120. We also tested whether co-immunization regimens involving rSV40 delivery of both IL-15 and gp120 augmented and/or accelerated SV40-mediated immune responses further.

## Methods

### Cell Lines

The murine mastocytoma cell line P815 (ATCC, Bethesda, MD, USA) was used, and maintained in culture with Dulbecco's Modified Eagle's medium (DMEM), supplemented with 10% newborn calf serum (NCS) (Gibco BRL/Life Technologies, Grand Island, NY, USA). COS-7 cells (ATCC, Bethesda, MD, USA), were used to expand stocks of recombinant SV(gp120) and SV(mIL-15) viruses. Cytotoxic lymphocytes were obtained from spleens of immunized mice, and cultured in RPMI-1640 (Gibco BRL/Life Technologies, Grand Island, NY, USA) supplemented with 10% NCS (RPMI-10). Rabbit kidney fibroblasts (RK13 cells) and CV-1 cells (African green monkey kidney cells) were obtained from ATCC (Bethesda, MD, USA). RK13 cells were used to propagate stocks of VCB41, a vaccinia virus vector carrying HIV-1NL4-3 envelope gp120 sequence.

### Mice

BALB/cJ mice aged 6–8 weeks were purchased from Jackson Laboratories, Bar Harbor, ME, USA. They were fed and housed in accordance with American Association for Accreditation of Laboratory Animal Care standards. Use of mice in the laboratory protocols described was approved by the Thomas Jefferson University Institutional Animal Care and Use Committee.

### Generation of SV(gp120)

A 1.6 kb DNA fragment encoding gp120 from HIV-1NL4-3 was made by PCR using primers with engineered restriction sites. This PCR product was cloned into pT7A5 (a plasmid containing an SV40 genome, in which large T antigen gene was replaced by cytomegalovirus (CMV) immediate early promoter and downstream polylinker), giving pT7A5-gp120. To make SV(gp120), the SV(gp120) genome was released from the carrier plasmid by restriction digestion, and used to make recombinant virus in COS-7 cells as described previously [34]. Virus stocks were purified and titered, as described elsewhere [34]. SV(HBS), a control virus for these studies, carries hepatitis B surface antigen (HBsAg), and has been reported previously [11].

### Generation of SV(mIL-15)

To generate a recombinant SV40 virus with the murine IL-15 transgene (SV(mIL-15)), mIL-15 cDNA was cloned into pSL-4p, which contains a *Tag*-deleted SV40 genome [[35], Vera, *et al*., in preparation], to yield prSVmIL-15. Virus was made from this plasmid in COS-7 cells as previously reported [32].

### Immunization of mice

Mice were given monthly 1 × 10^9 ^infectious units (IU) of SV(gp120) ± SV(mIL-15) intraperitoneally (IP). In some studies, final administrations included both IP and subcutaneous (SQ) inoculations. SV(HBS) was used as a control antigen-carrying vector. Specific immunization schedules are described in the **Results **section, below.

### Stably-transfected P815 target cells for cytotoxicity assays

Production of HIV Env-expressing stably transfected targets is similar to the procedure used for generating SIV Env-expressing targets [12]. Briefly, gp120 cDNA was cloned into pCDNA3. The resulting plasmid, pcgp120, was co-transfected into P815 cells together with the neomycin resistance-carrying plasmid, pSV2Neo. Transfected cells were selected in G418-supplemented DMEM-10, then cloned by limiting-dilution. Viable clones were expanded, assayed for gp120 expression by flow cytometry, and maintained thereafter in G418-supplemented medium.

### Flow cytometric detection of cell surface gp120 expression

Flow cytometry was used to verify gp120 expression on the surface of P815 cells. A recombinant vaccinia virus carrying HIV-1NL4-3 gp120 (VCB41, NIH AIDS Reference Reagent Repository Program (NIH-ARRRP)) was used both as a positive control for gp120 expression and also to generate gp120-expressing target cells in some experiments. Cells that had been stably-transfected with plasmid gp120, or infected with VCB41 both expressed gp120, as assayed by flow cytometry (Coulter-Epic, Kimmel Cancer Center, TJU) (data not shown).

The gp120-expressing P815 population was then cloned by limiting dilution. Clonal outgrowths were then reanalyzed by cytofluorimetry (FACS, data not shown) and the single clone expressing the highest levels of gp120, clone 24, was used in subsequent studies as a target for cytotoxicity assays.

### Anti-gp120 binding-antibody detection using a CELISA

An ELISA method was used to assay the activity of anti-gp120 antibodies elicited by immunization of the mice with SV(gp120) ± SV(mIL-15). The strategy for our appraoch to testing for antibodies vs. HIV Env is similar to one we have used to measure binding activity vs. SIV Env [12]. Briefly, a cell-based assay was developed using VCB41-infected P815 cells (cells were infected with virus for 48 hours prior to being used in assay) as control targets. Sera were taken from mice at 2- and 4-week intervals after immunization(s). Antibody reactivity vs. cell membrane-expressed gp120 was tested by measuring A_405nm _of test sera vs. VCB41-infected P815 cells, subtracting A_405nm _due to binding to wild type (wt) VV-infected P815 cells, and also subtracting A_405nm _of control sera from mice immunized in parallel with a control rSV40, SV(HBS)

### Measurement of cytotoxic lymphocyte activity by specific lysis of ^51^Cr-labeled target cells

Wild type (wt) P815 cells, or clone 24 P815 cells expressing gp120 were the target cells for unselected lymphocytes from spleens of mice immunized with SV(gp120) (± SV(mIL-15)), or SV(HBS) as control. Where SV(gp120) was used alone, P815 cells infected with wt vaccinia virus or VCB41 were target cells for spleen cells of immunized mice.

Mice were boosted simultaneously with 1 × 10^9 ^IU intraperitoneally (IP) and 1 × 10^8 ^IU subcutaneously (hind footpads) usually 4 d before assay, but up to 1 month prior to assay, to test cytolytic lymphocyte memory. Spleen cell concentrations adjusted to 2 × 10^6 ^/ ml with RPMI-10.

In some assays of cytotoxic lymphocyte memory, effector cells were harvested 1 month after the final injection. Effector cells from immunized mice were prepared as described, but in addition, were incubated with 5 μg/ml Concanavalin A (Con A, Sigma Chemical Co., St. Louis, MO) overnight, prior to assay. Con A-stimulated cells were then harvested, washed once in RPMI-10, and then used with target cells in the assay.

P815 target cells were washed, then labeled with ^51^Cr (ICN Biomedicals, Inc., Irvine, CA, USA) (100 μCi per 1 × 10^6 ^cells) at 37°C, 5% CO_2 _for 4 h as described previously [12]. Afterwards, target cells were washed, then plated in triplicate with effector cells (splenocytes) at effector:target (E:T) ratios of 20:1 and 10:1, and incubated at 37°C, 5% CO_2 _for 4 hours. Supernatant ^51^Cr was counted (1282 Compugamma CS, LKB) [12]. Mean specific lysis was calculated as:

Mean c.p.m. for gp120-immunized effector cells mixed with gp120-expressing targets, minus the mean c.p.m. control (SV(HBS))-immunized effector cells vs gp120-expressing targets, and expressed as a percentage of the maximal target cell lysis (target cells incubated with 1% Triton-X). Background release of ^51^Cr from wild type target cells was subtracted. Thus:

% specific ^51^Cr release = {[c.p.m. ^51^Cr released by SV(gp120)-immune populations from gp120-expressing P815 cells] minus [c.p.m. ^51^Cr released by SV(HBS)-immune lymphocytes from gp120-expressing P815 cells]}, divided by [c.p.m. ^51^Cr released by Triton X-100 from gp120-expressing P815 cells]. The same calculations were done for lysis of wild type P815 cells by gp120-immune and control-immune effector populations. These numbers were then subtracted from the calculated ^51^Cr release above to determine the gp120-specific lysis of target cells by SV(gp120)-immunized effector cells.

### Western analysis of IL-15 expression in SV(mIL-15)-transduced P815 cells

P815 cells were transduced with SV(mIL-15) ×1 at m.o.i. = 100. Culture supernatants were harvested at several times post-transduction, and stored at -80°C. At day 6 post-transduction, a well of cells was harvested and lysed (2% NP40, 50 mM Tris pH7.4,150 mM NaCl, 1 mM EDTA, 10% Glycerol + protease inhibitor cocktail (25× stock Complete™ EDTA-free protease inhibitor cocktail, Roche Diagnostics GmbH, Mannhein, Germany)). Remaining wells were activated non-specifically with 5 mg/ml Con A, and supernatants harvested at various times thereafter. 3 days after con A stimulation, cells were lysed as described above. 50 μg of each culture supernatant or lysate were loaded on a 4–20% Tris-HCl gradient gels (Ready Gel, Bio-Rad, Hercules, CA, USA). 50 ng recombinant human IL-15 was used as a positive control. Samples were electrophoresed, and blotted to PVDF membranes (Immobilon™-P, Millipore Corporation, Bedford, MA). Blots were blocked overnight at 4°C with 5% milk in PBS + Tween-20 (0.05%). Rabbit anti-mouse IL-15 (Abcam, Cambridge, UK) was used as primary antibody, (diluted 1:500 with PBS-Tween), for 2 h at 37°C. Horseradish peroxidase (HRP)-conjugated goat anti-rabbit IgG (Jackson ImmunoResearch, West Grove, PA) was used at 1:10,000 dilution in PBS-Tween, for 1 h at room temperature. Signal was detected with chemiluminescence reagent (ECL Plus, Amersham Pharmacia Biotech UK Ltd., Little Chalfont, UK,)

### Assaying for IFNγ production stimulated by IL-15

COS-1 cells were infected with SV(mIL15) or SVLUC (carrying luciferase) as a negative control. 24 h later, the media were changed and cells incubated 48 h in 500 μl of RPMI 10% serum/well. Fresh mouse spleen cells (5000/well) then cultured 48 h with 100 μl of cell supernatant + 100 μl of RPMI 10% serum. IFNγ ELISA (Pharmingen) was performed on the supernatant from these cultures.

## Results

### Stably transfected HIV-1 gp120-expressing P815 cells

P815 cells stably transfected to express HIVNL4-3 gp120 were selected and cloned by limiting dilution (see **Methods**). We used flow cytometry to identify the clone most strongly positive for cell membrane gp120. Compared to other stably-transfected clones, "clone 24" expressed gp120 at the cell membrane best (data not shown). VCB41-infected and SV(gp120)-transduced P815 cells also expressed substantial cell membrane gp120. Control wtP815 cells, or P815 cells infected with wt VV did not (data not shown).

Therefore, clone 24 cells were used to assay gp120-specific immune responses. In both antibody and cytotoxicity assays, two different types of background were subtracted from the responses of gp120-immunized mice: serum binding or cellular reactivity from gp120-immunized animals vs. wt P815 cells and reactivity from control (i.e., SV(HBS))-immunized rSV40-immunized mice vs. clone 24 cells. Thus, data presented below reflect gp120-specific responses against clone 24.

### Immunization with SV(gp120)

**Figure 1:**
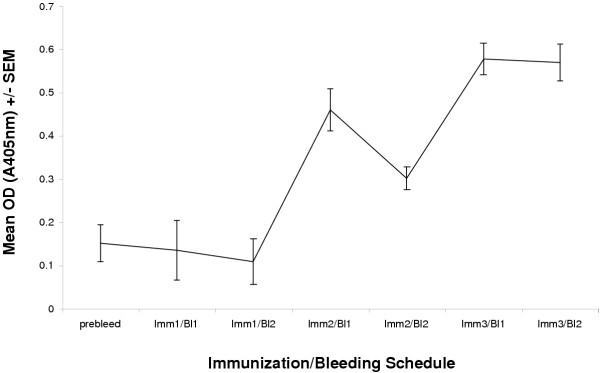
Serum antibody against HIV-1NL4-3 envelope glycoprotein gp120 in mice receiving multiple inoculations of SV(gp120) BALB/cJ mice were immunized at monthly intervals with 1 × 10^9 ^infectious units (IU) SV(gp120), IP. They were bled biweekly. Gp120-specific antibody reactivity was assayed by CELISA, as described in **Methods **and in reference #12. Specific binding of HIV-1 Env is shown here as specific A405nm, ± S.E.M.

Normal BALB/c mice were inoculated with SV(gp120), and their sera were assayed for reactivity vs. gp120 by CELISA. The details of this cell-based ELISA, or CELISA, as described in **Methods**. Specific binding antibody activity was first statistically significant, compared to prebleed sera 2 weeks after the second inoculation of SV(gp120) (P = 0.000332, using two-tailed Student's t-test) and reached a plateau after the third inoculation (P = 0.000000316 by the same analysis) (Figure 1). Additional immunizations beyond the third did not further increase detectable antibody levels (data not shown).

### Cytolytic responses against gp120: testing for cytotoxic lymphocyte memory

**Figure 2:**
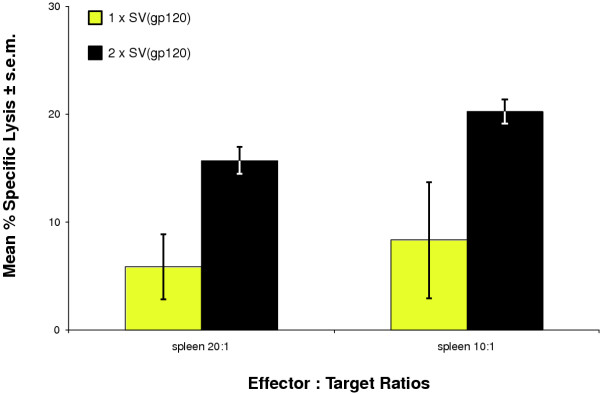
Specific cytolytic activity against HIV-1NL4-3 gp120 in mice immunized with SV(gp120) and assayed one month after final injection. BALB/cJ mice were immunized twice with SV(gp120) IP at monthly intervals. Splenocytes were harvested one month after final inoculation. Unselected effector cells were added to ^51^Cr-labelled target cells and specific lysis of gp120-expressing cells was calculated as described in **Methods**, ± S.E.M. Results shown here represent ≥ 3 independent determinations per data set.

An effective anti-lentiviral immunization regimen should generate cytotoxic memory cells. To see if SV(gp120) treatment could do this, mice were immunized once with SV(gp120) IP, then sacrificed 1 month later, without further treatment. In order to lyse target cells, committed cytotoxic cells require activation. However, to avoid antigen-specific selection and specific stimulation only of gp120-reactive cytotoxic cells, splenic lymphocytes were non-specifically stimulated by overnight incubation with Con A. A single immunization with SV(gp120) alone elicited only weak memory lytic responses (≤ 10% specific lysis) against gp120-expressing target cells (Figure 2).

A second group of animals received a second inoculation with SV(gp120) one month after the first, then were assayed the same way one month later for anti-gp120 cytolytic activity. These mice made stronger specific memory responses (15–20% specific lysis) than did animals given only a single inoculation (P ≤ 0.04, by Student's t-test, comparing 2 injections with just one) (Figure 2).

To test whether SV(gp120) could elicit very long term cytotoxic lymphocyte memory, mice were immunized monthly ×8 with SV(gp120) IP. A final IP inoculation with SV(gp120) was given *1 year after their eighth immunization*. They were sacrificed 4 days later, and direct gp120-specific splenocyte cytotoxicity was measured. Unselected spleen cells from all animals made very strong (≥ 50% specific lysis) gp120-specific cytolytic responses (mean specific lysis of 61% ± 4.2).

### IL-15 expression and secretion in SV(mIL-15)-transduced P815 cells

**Figure 3:**
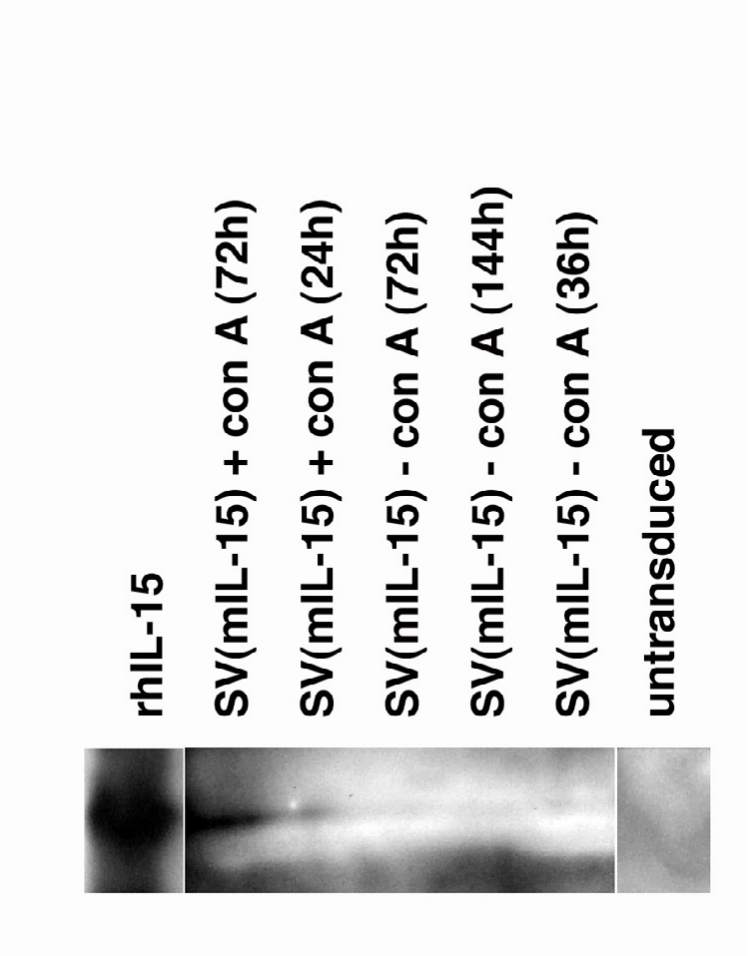
Western analysis of IL-15 expression in culture supernatants from SV(mIL-15)-transduced P815 cells P815 cells were transduced with SV(mIL-15) at a m.o.i. = 100. 1.5, 3 and 6 days post-transduction, culture supernatants were collected. Cultures were then stimulated with Con A and additional supernatants harvested 24 and 72 hours later. IL-15 secretion into supernatants was visualized by Western analysis. Recombinant human IL-15 (rhIL-15) was the positive control (non-adjacent lane). Supernatants from unstimulated cultures were tested in parallel (non-adjacent lane).

Because higher levels of durable memory cytotoxic responses could be achieved with repeated injections of SV(gp120), and lower levels were seen with 2 injections, we tried to accelerate development of such responses using IL-15, delivered by transduction. To determine if IL-15 could be expressed by transduction, P815 cells were transduced with SV(mIL-15) at m.o.i. = 100. Culture supernatants were harvested 36, 72 and 144 hours later, at which point the cultured cells were activated with Con A. Culture supernatants were collected at 24 and 72 hours post-activation. Supernatants were assayed for IL-15 secretion by Western analysis as described in **Methods**, using rabbit antibody vs. murine IL-15 (Figure 3). The positive control, recombinant human IL-15, has approximately 60% sequence homology with murine IL-15). IL-15 secretion was detectable, but just barely so, in unstimulated culture supernatants. It was abundant by 72 hrs post-stimulation. These data were used in planning co-transduction experiments.

The functionality of the IL-15 produced in this fashion was tested by exploiting the ability of IL-15 to elicit production of IFN-γ by lymphocytes. Thus, CV-1 and COS-7 cells (African green monkey kidney cells) were transduced by SV(mIL-15), then cultured for 72 hrs. Control cultures of the same cells were transduced with SVLUC (carrying luciferase as a transgene). Normal mouse spleen cells were cultured for 42 hrs in 200 μl of the resulting culture supernatants. Production of IFN-γ by the spleen cells was measured by ELISA. Supernatants from COS-7 and CV-1 cells elicited respectively 2056 ± 363 pg/ml and 880 ± 196 pg/ml IFN-γ. Supernatants from SVLUC-transduced cells did not elicit detectable interferon secretion by spleen cells (<20 pg/ml).

### Effects of IL-15 in cytotoxic lymphocyte responses against gp120

To determine whether coordinate administration of SV(mIL-15) plus SV(gp120) improved cytolytic responses against gp120, mice were given two sets of injections IP, one month apart. Normal BALB/c mice received IP with 10^9 ^IU of rSV40: one group was given SV(mIL-15) alone, and one group SV(gp120) alone. Three other groups received both SV(mIL-15) and SV(gp120): either SV(mIL-15) followed 3 days later by SV(gp120), or SV(gp120) first, followed by SV(mIL-15). The final group was given both SV(mIL-15) and SV(gp120) simultaneously. The 3 day separation between the two vectors was used because of the strength of the signal for secreted IL-15 by Western blotting at 72 hours post-stimulation (see above). One month later, unselected spleen cells were assayed as described above for cytolytic activity against gp120-expressing clone 24 cells.

**Figure 4:**
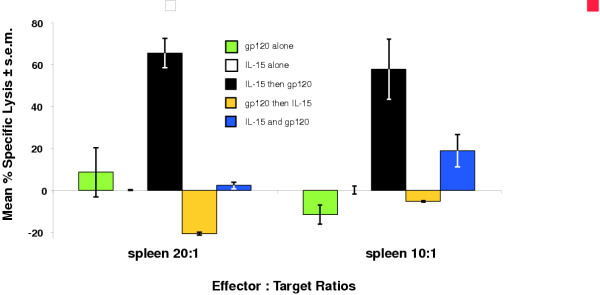
Specific cell-mediated responses against gp120-expressing target cells by splenic effectors from co-immunized mice Mice were given two monthly injections with either SV(mIL-15), SV(gp120) or both cytokine and antigen sequentially or simultaneously IP. One month after the final inoculation(s), unselected spleen cells were assayed for specific cytolytic activity against gp120-expressing clone 24 cells labeled with ^51^Cr, as described in **Methods**. Results shown here represent ≥ 3 independent determinations per data set.

Adding IL-15 to the immunization regimen greatly increased gp120-specific cytolytic responses (Figure 4). Also, the timing of cytokine administration relative to SV(gp120) inoculation significantly affected the responses seen. Mice given SV(mIL-15) 3 d after SV(gp120) did not make detectable gp120-specific cytotoxic responses. Simultaneous inoculation with SV(mIL-15) and SV(gp120), however, increased specific cytolysis to ≥ 20%, which was significant at E:T = 20:1 (P ≤ 0.05, using Student's t-test) compared to SV(gp120) alone. The most dramatic results were observed when SV(mIL-15) was administered 3 d before SV(gp120). Those mice demonstrated highly significant augmentation by SV(IL-15) of gp120-specific lysis, which was ≥ 60% at both 20:1 and 10:1 E:T ratios (P ≤ 0.02 using Students' t-test, compared with immunization of SV(gp120) alone). Mice injected ×2 with SV(mIL-15) alone made no significant gp120-specific cytolytic responses at either 20:1 or 10:1 effector:target ratios. Mice given only SV(gp120) demonstrated ≈ 10% specific lysis at E:T = 20:1.

### Effect of IL-15 co-administration on anti-gp120 antibody responses

Mice receiving SV(mIL-15) and/or SV(gp120) (or the control vector, SV(HBS)) according to the schedules outlined above were tested to determine the effect, if any, of such co-administration in anti-gp120 serum antibody responses. CELISA and calculation of gp120-specific antibody binding were performed as described in **Methods**.

Slight binding antibody activity was detected 2 weeks after the first inoculation(s), in all SV(gp120) recipient groups. Levels of SV(gp120)-induced groups made detectable antibody responses were not appreciably affected by coadministration of SV(mIL-15) (data not shown).

## Discussion

In this study, we used rSV40 vectors to elicit HIV-1NL4-3 gp120-specific cytotoxic lymphocyte and antibody responses. We have observed that these vectors may be administered repeatedly to boost those responses. Further studies also suggested that such responses are durable *in vivo*. Our results here demonstrate several important strengths of using rSV40 vectors to immunize against lentiviral antigens: Among these are the ability of the vector to be administered multiple times without eliciting neutralizing responses [11-13], and the magnitude of the cytotoxic responses to the vector-encoded lentiviral target antigen. When SV(gp120) was given alone, i.e. without added SV(mIL-15), levels of specific cytotoxicity increased with additional SV(gp120) injections: After 2 injections, ≈ 20% specific lysis was seen, which increased to >70% specific lysis after 7 injections. The potency of rSV40 immunization to elicit cytotoxic immune responses is underscored by the fact that these responses were measured in direct ^51^Cr-release assays: unselected lymphoid organ populations were added directly to labeled target cells at low E:T ratios, and specific ^51^Cr release was measured.

Analysis to confirm CD8 expression, or expression of other CTL markers was not performed on the effector cells. However, it is unlikely that these data reflect the cytotoxic activity of NK cells. NK cytolytic activity is non-specific and does not increase with repeated immunization. The patterns of ^51^Cr release observed in the current studies were extensively controlled to ascertain the antigen-specificity of the cytolysis observed: background lysis of wild type P815 cells was subtracted, as was lysis by lymphocytes from mice immunized with an irrelevant rSV40 vector. We also found that cytolysis increased with increasing numbers of immunizations, which is not a characteristic of NK cell-mediated lysis.

Since a key goal for a vaccine against HIV is to generate immune responses that are durable *in vivo*, we tested whether cytotoxic lymphocyte activity elicited by SV(gp120) immunization, was detectable one month after inoculation. Thus, cytolytic responses, assayed one month after a second injection, were ≈ 20%, which is comparable to those of splenic cytotoxic cells assayed four days following a third inoculation (data not shown). Further, mice given multiple injections of SV(gp120), then rested *for one year, *gave ≈ 70% specific lysis when challenged with SV(gp120). Therefore, SV(gp120) administration may thus favor development of cytotoxic lymphocyte memory.

In an attempt to accelerate and to improve upon these specific cytotoxic and particularly cytotoxic memory responses, we co-immunized mice with SV(gp120) and a rSV40 carrying mouse IL-15. IL-15 promotes cytotoxic lymphocyte responses, and in particular, cytotoxic memory responses [23,24]. The biological effects of IL-15 are less well understood than are those of some of the other immunostimulatory cytokines that have been applied to these types of immunization protocols, such as IL-2, IL-12 and IFN-γ. IL-15 is not a T cell-derived product, but rather appears to be produced by a variety of cells, such as epithelial cells, stromal cells and muscle. It acts on activated T cells, sometimes similarly to IL-2, but it has activities distinct from those of IL-2. IL-15 may play a role in T cell activation in the CNS. It also promotes cytotoxic responses, cytotoxic T cell memory, and natural killer (NK) cell maturation [33,34].

Accordingly, our analysis of the contribution by IL-15 to cytotoxic responses, focused mainly on the ability of SV(mIL-15) to augment specific cytotoxic responses of spleen cells from animals rested 1 month following immunization. Because quiescent cytotoxic T cells are not strong effectors, we non-specifically activated the splenocytes prior to assay with Con A. Non-specific activation was used to avoid specifically enriching effector cell population for gp120-specific cells *in vitro*. Furthermore, low effector:target ratios (20:1 and 10:1) were used in these assays. Our immunization protocols tested both simultaneous and staggered administration of rSV40s carrying HIV-1NL4-3 gp120 and murine IL-15.

IL-15 co-immunization dramatically accelerated cytotoxic responses, depending on the immunization regimen used: Animals given SV(mIL-15) alone made no gp120-specific cytolytic responses. Mice receiving 2 treatments with a mixture of SV(gp120) and SV(mIL-15) gave much higher specific lysis, depending on the coadministration regimen, as compared to those receiving SV(gp120) alone (≈ 10% specific lysis). Thus, among mice given staggered injections of SV(mIL-15) and SV(gp120), the order of cytokine administration greatly affected the response: if SV(gp120)was given first, no detectable gp120-specific cytolysis was observed. However, if the cytokine was given first, followed 3 days later by SV(gp120), ≥ 60% specific lysis was seen at both 20:1 and 10:1 effector:target ratios.

Why the order of cytokine administration should affect antigen-specific responses so dramatically is not yet clear. Cytokine given after, or together with antigen, may have insufficient time to augment cytotoxic responses. In addition, Western analysis of IL-15 production showed that IL-15 secretion was not detectable in supernatants beyond 36 hours, but could be stimulated subsequently. Thus, a specific, possibly brief, window for IL-15 expression and secretion may need to be attained, in order for its effects on gp120-specific responses to be detectable. We observed very high levels of specific lysis by these unselected effector populations following just two tandem injections of SV(mIL-15) followed by SV(gp120).

The strong anti-lentiviral cytolytic responses we report were observed in a strain of mouse, BALB/cJ, that generally mounts relatively weak type 1 T cell responses. The finding of >60% cytolysis with two administrations of SV(gp120) + SV(mIL-15), suggests that a strategy similar to that described herein may be helpful in individuals who would generate relatively low cytolytic responses.

Serum antibody levels assayed by CELISA where SV(gp120) was administered alone, multiple times, were detectable after two immunizations, and continued to increase up to week 4 following the third immunization. These responses were not further enhanced by subsequent boosting immunizations. While specific antibody responses against gp120 were detected in all experimental groups following SV(gp120) and SV(mIL-15) co-immunization, IL-15 co-administration did not augment anti-gp120 antibody levels, compared to gp120 alone. This was to be expected, since IL-15 reportedly acts primarily on T cell and NK cell functions, rather than on humoral immune responses.

Our data argue in favor of using IL-15 as an adjuvant for antigen-specific immune responses, particularly cytotoxic lymphocyte responses. We also demonstrate that a single transgene, administered multiple times (>3), may be very effective at eliciting both humoral and cell-mediated responses. These results thus both corroborate and extend our previous observations [13,14,32], and suggest that combining rSV40s encoding antigens and immunostimulatory cytokines sequentially in multi-administration regimens may provide high levels of long-lasting immunity against the target antigen.

## Conclusions

Recombinant SV40-derived gene-delivery vectors, being transparent to the immune system, can be given multiple times to prime and boost immune responses against the delivered antigens. Anti-vector immunity does not overwhelm responses against the target antigens. As well, these vectors elicit very high levels of antibody, and especially cell-meditated immunity. Finally, combining the delivery of rSV40s bearing antigens with those bearing cytokines such as IL-15 can enhance levels of immunity, particularly long-term immunity. Clearly, much work remains. However, this approach offers promise as a strategy to immunize against pathogens for which classical approaches have not been adequately effective.

## List of Non-Standard Abbreviations Used

**Table 1:** 

**Abbreviation**	**Meaning**
CELISA	cell-based ELISA
E:T	effector cell:target cell ratio
gp120	major HIV envelope glycoprotein, 120 kDa
HBS	hepatitis B surface antigen
IFNγ	interferon-gamma
mIL-15	mouse interleukin-15
NIH-ARRRP	National Institutes of Health, AIDS Research Reference Reagent Program
pNPP	p-nitrophenyl phosphate
VCB41	strain of vaccinia virus carrying gp120 coding sequences
wt	wild type

## Competing Interests

None declared.

## Authors' Contributions

HJM devised all the assay systems for cell- and antibody-mediated immunity against lentiviral antigens, performed all the immunization studies and assays. HM also wrote this manuscript. PYT generated the SV(gp120) construct. MV and PF generated the SV(mIL-15) and SVLUC constructs described here and performed the ELISA for IFNγ stimulated by SV(mIL-15). DSS is the Principal Investigator for this work, oversaw and planned the experimental strategies, worked with HJM in interpreting the experimental data and writing the manuscript.
